# Autophagy dictates sensitivity to PRMT5 inhibitor in breast cancer

**DOI:** 10.1038/s41598-023-37706-9

**Published:** 2023-07-03

**Authors:** Charles Brobbey, Shasha Yin, Liu Liu, Lauren E. Ball, Philip H. Howe, Joe R. Delaney, Wenjian Gan

**Affiliations:** 1 Department of Biochemistry and Molecular Biology, Medical University of South Carolina, Charleston, SC 29425 USA; 2 Department of Cell and Molecular Pharmacology and Experimental Therapeutics, Medical University of South Carolina, Charleston, SC USA

**Keywords:** Biochemistry, Cancer, Molecular biology

## Abstract

Protein arginine methyltransferase 5 (PRMT5) catalyzes mono-methylation and symmetric di-methylation on arginine residues and has emerged as a potential antitumor target with inhibitors being tested in clinical trials. However, it remains unknown how the efficacy of PRMT5 inhibitors is regulated. Here we report that autophagy blockage enhances cellular sensitivity to PRMT5 inhibitor in triple negative breast cancer cells. Genetic ablation or pharmacological inhibition of PRMT5 triggers cytoprotective autophagy. Mechanistically, PRMT5 catalyzes monomethylation of ULK1 at R532 to suppress ULK1 activation, leading to attenuation of autophagy. As a result, ULK1 inhibition blocks PRMT5 deficiency-induced autophagy and sensitizes cells to PRMT5 inhibitor. Our study not only identifies autophagy as an inducible factor that dictates cellular sensitivity to PRMT5 inhibitor, but also unearths a critical molecular mechanism by which PRMT5 regulates autophagy through methylating ULK1, providing a rationale for the combination of PRMT5 and autophagy inhibitors in cancer therapy.

## Introduction

Arginine methylation has emerged as one of the common posttranslational modifications (PTMs) and plays crucial roles in controlling protein stability, localization, protein–protein interaction, and enzymatic activity^[1–4]^. Protein arginine methyltransferases (PRMTs) serve as writers to catalyze the transfer of methyl groups to arginine residues, thereby generating three types of methylarginines: monomethylarginines (MMA), asymmetric dimethylarginines (aDMA), and symmetric dimethylarginines (sDMA). In mammals, nine PRMTs are grouped into three categories based on their products: type I PRMTs (PRMT1, PRMT2, PRMT3, PRMT4, PRMT6, and PRMT8) catalyze the formation of MMA and aDMA, while type II PRMTs (PRMT5 and PRMT9) register MMA and sDMA. PRMT7 is the sole member of type III enzyme that generates only MMA^[5,6]^. Dysregulation of PRMTs has been associated with many human diseases and has become attractive therapeutic targets^[7–10]^.

PRMT5 is the main type II enzyme with numerous substrates that are involved in fundamental cellular processes^[11]^. PRMT5 regulates transcription by depositing sDMA on histone (H4R3, H3R8, H3R2, and H2AR3)^[12–14]^ and transcription factors^[15–17]^, while it controls DNA damage response in both transcriptional-dependent and -independent mechanisms^[18–20]^. PRMT5 is also a critical regulator of RNA splicing^[21,22]^ and cell signaling transduction^[23–25]^. Clinically, PRMT5 overexpression has been observed in a variety of cancers^[26,27]^. Moreover, elevated PRMT5 expression is associated with poor prognosis and chemotherapeutic resistance in breast cancer patients^[28,29]^. Multiple PRMT5 inhibitors have been developed and are currently being evaluated in clinical trials^[30]^. Interestingly, preclinical studies showed that breast cancer cells display diverse sensitivity to PRMT5 inhibitors with triple negative breast cancer (TNBC) cells generally being relatively resistant^[31,32]^. However, the underlying causes for this variation in sensitivity to PRMT5 inhibitors remain elusive.

Autophagy is a self-degradative process that delivers cytoplasmic materials to lysosomes for degradation and serves as a key recycling factory to maintain cellular homeostasis^[33,34]^. The autophagy process includes five sequential steps: initiation, nucleation, elongation/expansion, autophagosome fusion, and degradation in autolysosome. Each step is executed by distinct complexes that are formed by evolutionarily conserved autophagy-related (ATG) proteins^[35–37]^. For example, the ULK kinase complex, which is composed of ULK1 or ULK2, ATG13, FIP200, and ATG101, is required for the initiation of autophagy^[38]^, while the class III PI3K complex I consisting of VPS34, VPS15, and Beclin 1 is essential for nucleation^[39]^. During autophagy, the ATG4-processed form of LC3 (LC3-I) is further converted to the PE-conjugated form (LC3-II) by ATG7-ATG3^[40,41]^, while the adaptor proteins p62/SQSTM1 is degraded^[42,43]^. LC3-II accumulation and p62 degradation are widely accepted as autophagy markers^[44]^.

Dysregulation of autophagy has been linked to various human diseases, particularly neurodegenerative diseases and cancers^[45]^. Autophagy has both tumor suppressive and tumor promoting roles depending on cancer types and stages^[46]^. Moreover, autophagy can be induced by therapeutic agents and plays a prosurvival role to confer drug resistance^[47,48]^. Therefore, targeting autophagy is a promising strategy to enhance therapeutic efficacy. Indeed, combination of autophagy inhibitors with different cancer treatments is currently undergoing evaluation in numerous clinical trials^[49]^.

In this study we demonstrate that PRMT5 inhibition induces cytoprotective autophagy and thereby decreases sensitivity to PRMT5 inhibitor in TNBC cells, and that PRMT5 negatively regulates autophagy in part by methylating ULK1. Thus, our study provides a molecular basis and rational for targeting both PRMT5 and autophagy as a potential option for TNBC treatment.

## Results

### Autophagy blockage sensitizes TNBC cells to PRMT5 inhibitor

**Figure 1:**
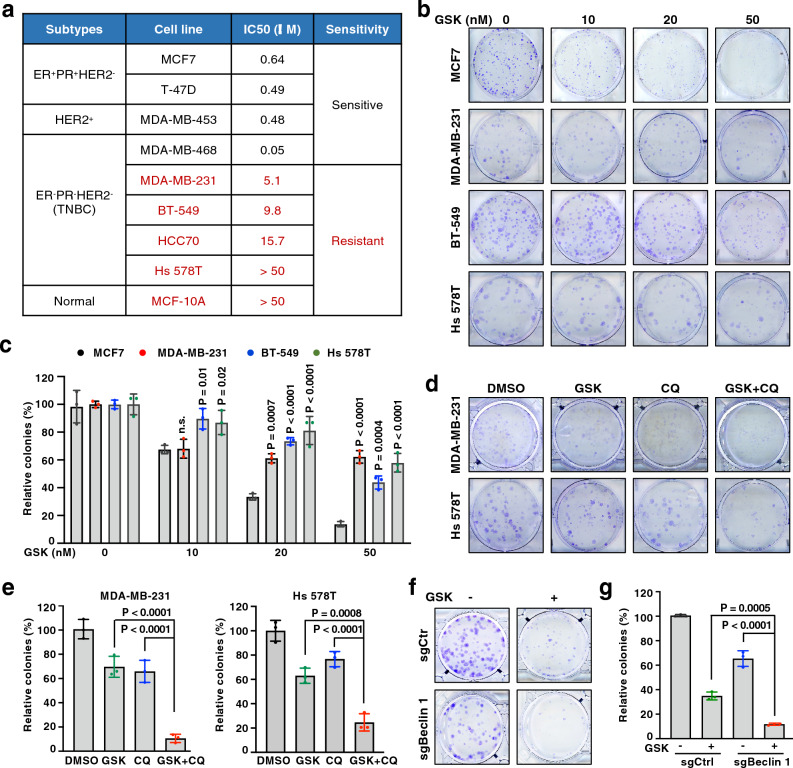
Autophagy inhibition sensitizes breast cancer cells to PRMT5 inhibitors. (**a**) IC50 of various breast cancer cell lines determined by cell viability assays. Cells were treated with GSK3326595 at 0, 50 nM, 500 nM, 1 μM, 5 μM, and 50 μM for 4 days before measuring cell viability. (**b**,** c**) Cells were treated with GSK3326595 (GSK) at indicated doses and subjected to colony formation assays. Representative images are shown in (**b**), and relative colony numbers are plotted in (**c**). (**d**,** e**) MDA-MB-231 and Hs 578T cells were treated with DMSO, GSK, chloroquine (CQ) or both and subjected to colony formation assays. MDA-MB-231, 50 nM GSK and 5 μM CQ; Hs 578T, 50 nM GSK and 2 μM CQ. Representative images are shown in (**d**), and relative colony numbers are plotted in (**e**). (**f**,** g**) BT-549 cells were depleted of Beclin 1 by sgRNA (sgCtr as a control) and then treated with 50 nM GSK and subjected to colony formation assays. Representative images are shown in (**f**), and relative colony numbers are plotted in (**g**). In (**c**,** e**,** g**), data are shown as the mean ± SD of n = 3 independent experiments. *P* values were calculated by Student’s *t* test.

Although PRMT5 inhibitors are currently being tested in clinical trial, it remains an open question whether they will be effective, and whether resistance will arise. To provide evidence for this knowledge gap, we evaluated the sensitivity of breast cancer cells to a specific PRMT5 inhibitor, GSK3326595 that has been tested in a phase II clinical trial for breast cancer (NCT04676516)^[31]^. We observed that ER^+^PR^+^ and HER2^+^ breast cancer cells, and one TNBC cell line (MDA-MB-468) were sensitive to GSK3326595, whereas the other four TNBC cell lines and a widely used non-malignant breast epithelial cell line (MCF10A) were relatively resistant to GSK3326595, which was defined by IC_50_ < 4 μM and IC_50_ > 4 μM as previously described^[32]^ (Fig. 1a and Supplementary Fig. 1a). We also performed colony formation assay to confirm this resistant phenotype in TNBC cells (Fig. 1b, c). Thus, understanding the molecular mechanisms of this observed resistance to PRMT5 inhibitors will benefit TNBC patients from treatment with PRMT5 inhibitors.

Autophagy can serve as a critical survival mechanism behind drug resistance and has been shown to protect breast cancer cells from death in response to certain chemotherapies and targeted therapy^[47,48]^. We reasoned that cytoprotective autophagy contributes to the observed resistance to PRMT5 inhibitor in TNBC cells. To test this idea, we treated TNBC cells with GSK3326595 and chloroquine (CQ), the only FDA-approved autophagy inhibitor that functions by preventing lysosomal degradation^[50]^. By treating cells with different doses of CQ, we determined cell line-specific doses that were used in the combination treatment (Supplementary Fig. 1b). Notably, CQ treatment increased GSK3326595-induced cell death in resistant TNBC cells (Supplementary Fig. 1c). Consistently, TNBC cell lines displayed different sensitivity to CQ in colony formation assays (Supplementary Fig. 1d–g). A combination of GSK3326595 with CQ significantly reduced colony formation, compared to single agent (Fig. 1d, e and Supplementary Fig. 1h, i). Consistent with these findings, we also observed an enhanced cytotoxic effect of GSK3326595 in Beclin 1-depleted cells (Fig. 1f, g). Given that PRMT5 inhibitors suppress cell proliferation in part by promoting apoptosis^[31]^, we found that co-treatment of GSK3326595 and CQ led to a marked increase of cleaved caspase 3 (Supplementary Fig. 1j), one of the best-known apoptotic markers^[51]^. These results demonstrate that autophagy blockage sensitizes resistant TNBC cells to PRMT5 inhibitor.

### Deficiency in PRMT5 induces autophagosome formation

**Figure 2:**
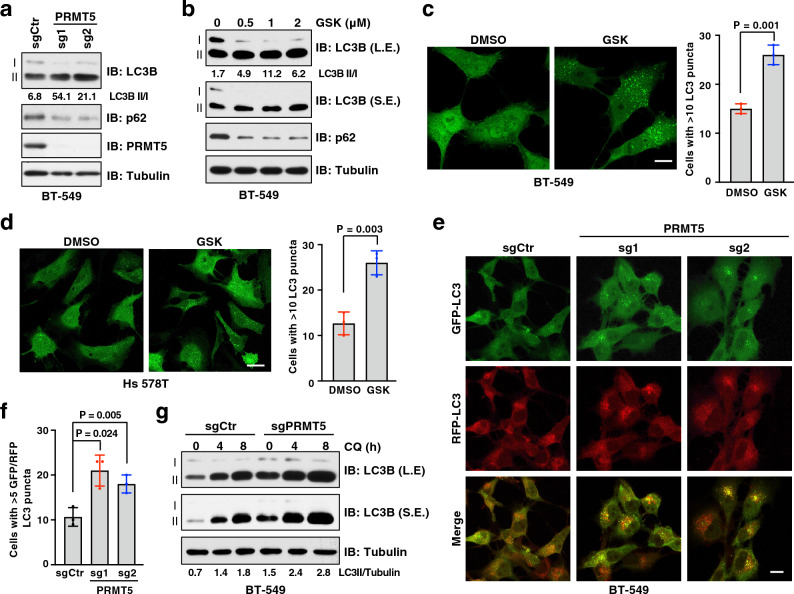
Inhibition of PRMT5 induces autophagy. (**a**) Immunoblot (IB) analysis of whole cell lysates (WCL) derived from BT-549 cells depleted of PRMT5 by two independent sgRNAs. (**b**) IB of WCL derived from BT-549 cells treated with GSK3326595 (GSK) at indicated doses for 3 days. (**c, d**) Representative images of GFP-LC3 puncta and cells with more than 10 puncta were counted in BT-549 (**c**) and Hs 578T (**d**) cells treated with DMSO or 1 μM GSK for 3 days. Scale bar, 10 μm. Data are shown as mean ± SD of n = 3 independent experiments with a total of 50 cells counted per experiment. *P* values were calculated by Student’s *t* test. (**e**, **f**) Representative images of GFP-LC3-RFP puncta in BT-549 cells depleted of PRMT5. Scale bar, 10 μm. Cells with more than 5 GFP-LC3 and RFP-LC3 puncta were counted as positive and data are shown as mean ± SD of n = 3 independent experiments with a total of 100 cells counted per experiment. *P* values were calculated by Student’s *t* test. (**g**) IB analysis of WCL derived from BT-549 cells depleted of PRMT5. Cells were treated with chloroquine 20 μM (CQ) for 0, 4, 8 h before harvesting. Similar results were obtained in n ≥ 3 independent experiments in (**a**, **b**, **g**).

To explore whether PRMT5 inhibition induces cytoprotective autophagy, we knocked out PRMT5 using CRISPR/Cas9 gene editing in multiple breast cancer lines and evaluated autophagy activity. Strikingly, depletion of PRMT5 led to an elevation of LC3-II/I ratio and a reduction of p62 protein levels under normal culture condition (Fig. 2a and Supplementary Fig. 2a, b). Consistently, treatment of cells with GSK3326595 increased autophagy activity (Fig. 2b and Supplementary Fig. 2c). To further support these immunoblot results, we monitored autophagy activity using the GFP-LC3 report system^[52]^ and found that there was a significant increase of GFP-LC3 puncta in GSK3326595-treated cells (Fig. 2c, d). Consistently, cells expressing the enzymatically dead mutant PRMT5-E444Q^[53]^ also enhanced LC3-II accumulation and p62 degradation, compared to cells expressing PRMT5-WT (Supplementary Fig. 2d), suggesting that PRMT5 regulates basal autophagy in a enzymatic-dependent manner.

Given that autophagy is a key biological process for adaptation to various stress events, such as nutrient deprivation, we next investigated whether PRMT5 is also involved in stress-induced autophagy. To this end, we found that compared to control cells, PRMT5-depleted cells displayed an additive LC3-II accumulation in response to the starvation of amino acids (Supplementary Fig. 2e). In contrast, overexpression of the PRMT5-E444Q mutant enhanced autophagy in the absence of amino acids, compared to overexpression of PRMT5-WT (Supplementary Fig. 2f). Previous studies have demonstrated that mTORC1-mediated phosphorylation of ULK1 at S757 is a key switch of autophagy induction in response to stresses^[54,55]^. Interestingly, we did not observe a significant difference on phosphorylation of ULK1-S757 between PRMT5-WT and PRMT5-E444Q expressing cells (Supplementary Fig. 2f). These results indicate that PRMT5-mediated regulation of autophagy is likely independent of the mTORC1 pathway and has an additive effect on nutrient deficiency-induced autophagy.

Of note, the accumulation of LC3-II in PRMT5-deficient cells could be caused by either enhanced LC3-I conversion to LC3-II or impaired LC3-II degradation^[56]^. To distinguish these two scenarios, we measured the autophagic flux using the mRFP-GFP-LC3 reporter system, which is based on the principle that GFP, but not mRFP, is quenched in the acidic environment, such as lysosome^[57]^. An increase of yellow (RFP^+^/GFP^+^) and red (RFP^+^) puncta indicates enhanced autophagosome formation, while only accumulation of yellow puncta suggests impairment in autophagosome-lysosome fusion and degradation. Notably, a significant accumulation of both yellow and red puncta of LC3 was observed in PRMT5-depleted cells (Fig. 2e, f). Moreover, treatment of cells with chloroquine (CQ), which inhibits autophagic flux by blocking autophagosome-lysosome fusion^[50]^, led to a further accumulation of LC3-II in PRMT5-depleted cells (Fig. 2g). These results suggest that PRMT5 suppresses autophagosome formation, but not autophagosome-lysosome fusion.

### ULK1 is required for PRMT5-mediated regulation of autophagy

**Figure 3:**
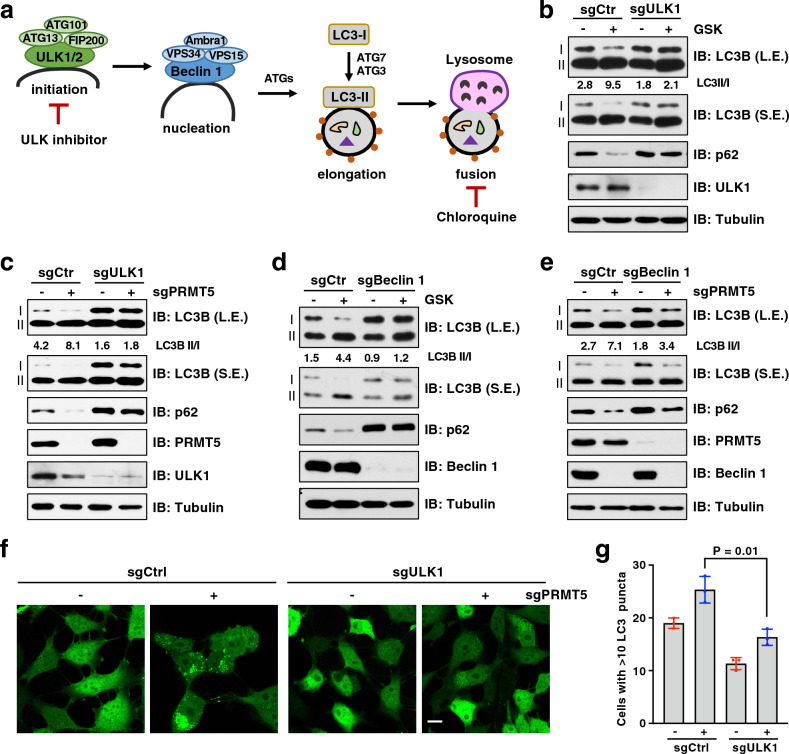
Depletion of ULK1 or Beclin 1 blocks PRMT5 inhibition-induced autophagy. (**a**) Schematic summary of the autophagy process highlighting some key components in the pathway. (**b**) IB analysis of WCL derived from BT-549 cells depleted of ULK1 by sgRNA. Cells were treated with 1 μM GSK3326595 (GSK) for 3 days before harvesting. (**c**) IB analysis of WCL derived from BT-549 cells depleted of ULK1 and/or PRMT5 by sgRNA. (**d**) IB analysis of WCL derived from BT-549 cells depleted of Beclin 1 by sgRNA. Cells were treated with 1 μM GSK3326595 (GSK) for 3 days before harvesting. (**e**) IB analysis of WCL derived from BT-549 cells depleted of Beclin 1 and/or PRMT5 by sgRNA. (**f**, **g**) Representative images of GFP-LC3 puncta in BT-549 cells depleted of ULK1 and/or PRMT5 by sgRNA. Scale bar, 10 μm. Cells with more than 10 puncta were counted as positive and data are shown as mean ± SD of n = 3 independent experiments with a total of 50 cells counted per experiment. *P* values were calculated by Student’s *t* test. Similar results were obtained in n ≥ 3 independent experiments in (**b**, **c**, **d**,** e**).

To investigate whether autophagosome formation induced by PRMT5 deficiency depends on the canonical autophagy pathway, we genetically ablated the core ATG genes involved in the initiation and nucleation stages (Fig. 3a). Strikingly, depletion of ULK1 largely blocked the induction of LC3-II in GSK3326595-treated or PRMT5-depleted cells (Fig. 3b, c). Moreover, ablation of ATG13, a component that enhances ULK1 activity and stability^[38]^, phenocopied the effects of ULK1 depletion (Supplementary Fig. 3). Furthermore, depletion of Beclin 1 that mediates nucleation downstream of ULK1 led to the blockage of autophagy induced by PRMT5 inhibitor or PRMT5 depletion (Fig. 3d, e). We also confirmed the immunoblot results using GFP-LC3 system and found that ULK1 depletion strongly decreased the formation of GFP-LC3 puncta in PRMT5-depleted cells (Fig. 3f, g). These results suggest that PRMT5 regulates autophagy in part through ULK1.

### PRMT5 interacts and methylates ULK1 at Arg532

**Figure 4:**
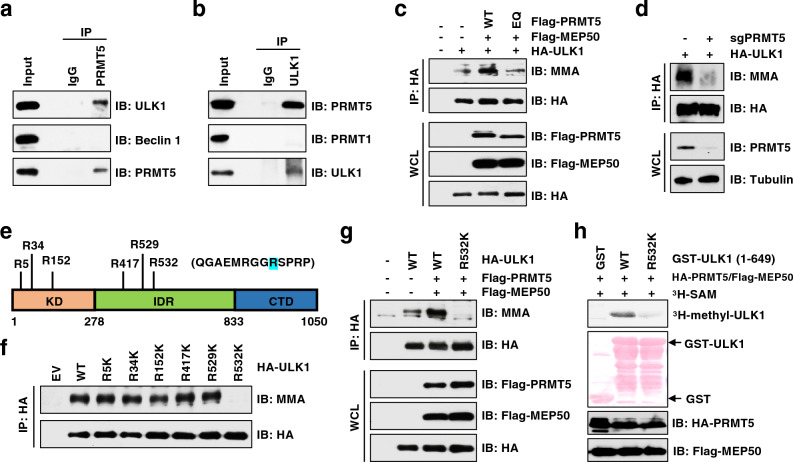
PRMT5 methylates ULK1 at Arg532. (**a**, **b**) IB analysis of WCL and immunoprecipitation (IP) products derived from MDA-MB-231 cells. IgG was used as a negative control. (**c**) IB analysis of WCL and IP products derived from HEK293T cells transfected with indicated constructs. (**d**) IB analysis of WCL and IP products derived from MDA-MB-231 cells stably expressing HA tagged ULK1 and infected with sgPRMT5 or sgCtr virus. (**e**) Schematic presentation of the putative methylated residues of ULK1. (**f**,** g**) IB analysis of WCL and IP products derived from HEK293T cells transfected with indicated constructs. (**h**) In vitro methylation of ULK1 in the presence of ^3^H-SAM. Truncated GST-ULK1 (1–649 aa) protein was purified from bacteria and HA-PRMT5/Flag-MEP50 were immunoprecipitated from HEK293T cells. Similar results were obtained in n ≥ 3 independent experiments in (**a**-**d**, **f**–**h**).

A recent study on ULK1 interactome identified PRMT5 as a partner of ULK1^[58]^. We speculated that this interaction plays a role in PRMT5-mediated regulation of autophagy. Consistent with the proteomic study^[58]^, we found that PRMT5 specifically co-immunoprecipitated endogenous ULK1, but not Beclin 1 (Fig. 4a). Reciprocally, ULK1 interacted with PRMT5, but not PRMT1 (Fig. 4b and Supplementary Fig. 4a). ULK1 contains an N-terminal kinase domain (KD), intrinsically disordered region (IDR) that is modified by multiple kinases for regulation of ULK1 activation, and a C-terminal early autophagy tethering (EAT) domain that is responsible for recruitment of ATG13, FIP200, and ATG101^[59]^. We found that PRMT5 specifically bound to the KD of ULK1 (Supplementary Fig. 4b), depletion of which abolished their interactions (Supplementary Fig. 4c). These results demonstrate that the KD is necessary and sufficient for ULK1 binding to PRMT5, which is distinct from ULK1 interaction with its known partners (Supplementary Fig. 4d).

Next, we investigated whether ULK1 is a substrate of PRMT5. Immunoblot analysis using an antibody against pan MMA^[60]^ showed that ULK1 was monomethylated (Supplementary Fig. 4e). Overexpression of PRMT5-WT, but not the enzymatically dead mutant PRMT5-E444Q, promoted MMA formation of ULK1 (Fig. 4c). In contrast, the MMA levels of ULK1 were severely decreased upon PRMT5 depletion (Fig. 4d). To identify which residue(s) is methylated by PRMT5, we analyzed ULK1 protein sequence by arginine methylation prediction tool, GPS-MSP^[61]^. Six arginine residues that were ranked top score were selected for further analyses (Fig. 4e). Notably, the R532K mutation, but not other mutations, abolished PRMT5-mediated MMA formation of ULK1 in cells (Fig. 4f, g). To demonstrate that PRMT5 directly methylates ULK1 at R532, we performed in vitro arginine methylation assays^[62]^ using the recombinant GST-ULK1 truncated protein that encompasses R532 (1–649 aa). Consistent with the finding in cells (Fig. 4g), the R532K mutant largely blocked PRMT5-mediated methylation in vitro (Fig. 4h). Although no SDMA signal was detected by immunoblot using the pan anti-SDMA antibody^[60]^, we could not rule out SDMA modification on ULK1-R532 because it might not be recognized by this antibody. Indeed, we identified dimethylation of ULK1 at R532 by mass spectrometry (Supplementary Fig. 4f). It warrants future development of the antibody that specifically recognizes symmetric dimethylation of ULK1-R532. Taken together, these results demonstrate that PRMT5 is the major physiological methyltransferases responsible for methylation of ULK1 on R532.

Interestingly, posttranslational modifications of ULK1, including phosphorylation by mTOR/AMPK^[54,63]^ and acetylation by TIP60^[64]^, were generally regulated by stresses. However, neither ULK1 MMA nor interaction between PRMT5 and ULK1 was affected in response to amino acid deprivation (Supplementary Fig. 4g, h), arguing that PRMT5-mediated regulation of ULK1 is independent of stress, at least nutrient deficiency.

### Blockage of ULK1-R532 methylation enhances ULK1 kinase activity and autophagy

**Figure 5:**
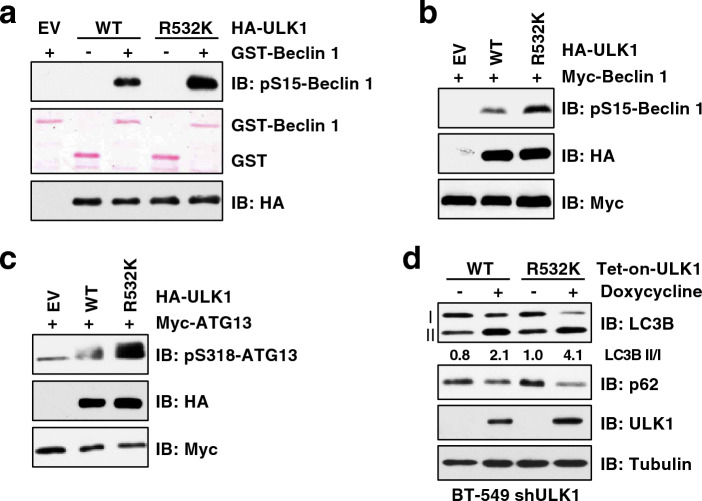
Methylation of ULK1 at Arg532 attenuates its kinase activity. (**a**) In vitro ULK1 kinase was performed using recombinant GST-Beclin1 (1–86 aa) purified from bacteria and HA-ULK1 immunoprecipitated from HEK293T cells. (**b**, **c**) IB analysis of WCL derived from HEK293T cells co-transfected with ULK1 and Beclin 1 (**b**) or ATG13 (**c**). (**d**) IB analysis of WCL derived from BT-549 cells depleted of endogenous ULK1 and re-expressing doxycycline inducible ULK1-WT or ULK1-R532K. Cells were treated with 1 μg/ml doxycycline for 8 h before harvesting. Similar results were obtained in n ≥ 3 independent experiments in (**a**–**d**).

Having established that ULK1 is methylated by PRMT5, we interrogated how this methylation affects its autophagic function. ULK1 phosphorylates multiple substrates to initiate autophagy process, such as Beclin 1 (Ser15)^[65]^ and ATG13 (Ser318)^[66]^. In vitro kinase assay showed that ULK1-R532K displayed higher kinase activity than ULK1-WT towards phosphorylating Beclin 1 (Fig. 5a). Moreover, compared to ULK1-WT, ULK1-R532K increased the phosphorylation of Beclin 1 and ATG13 in cells (Fig. 5b, c and Supplementary Fig. 5a). In an agreement of the enhanced activity of ULK1-R532K, cells expressing ULK1-R532K mutant exhibited an increased ratio of LC3-II/I and degradation of p62, compared to cells expressing ULK1-WT (Fig. 5d). These results suggest that PRMT5-mdediated ULK1-R532 methylation decreases its kinase activity to attenuate autophagy.

Next, we sought to investigate how ULK1-R532K enhances its kinase activity. Both ULK1-WT and ULK1-R532K bound to FIP200 and ATG13 at a comparable level (Supplementary Fig. 5b, c), indicating the ULK complex formation was not affected. We also did not observe a change of ULK1-R532K binding to its substrates, Beclin 1 and Ambra 1 (Supplementary Fig. 5c, d). Moreover, the interaction between ULK1-R532K and AMPK or Raptor (an essential subunit of mTORC1) was not significantly changed, compared to ULK1-WT (Supplementary Fig. 5e), further supporting the notion that R532 methylation regulates ULK1 activation is independent of or parallel to the mTORC1/AMPK pathway. These results suggest that ULK1-R532 methylation impairs its kinase activity unlikely through modulating ULK1 interactions with its partners.

### ULK inhibitor sensitizes resistant TNBC cells to PRMT5 inhibitor

**Figure 6:**
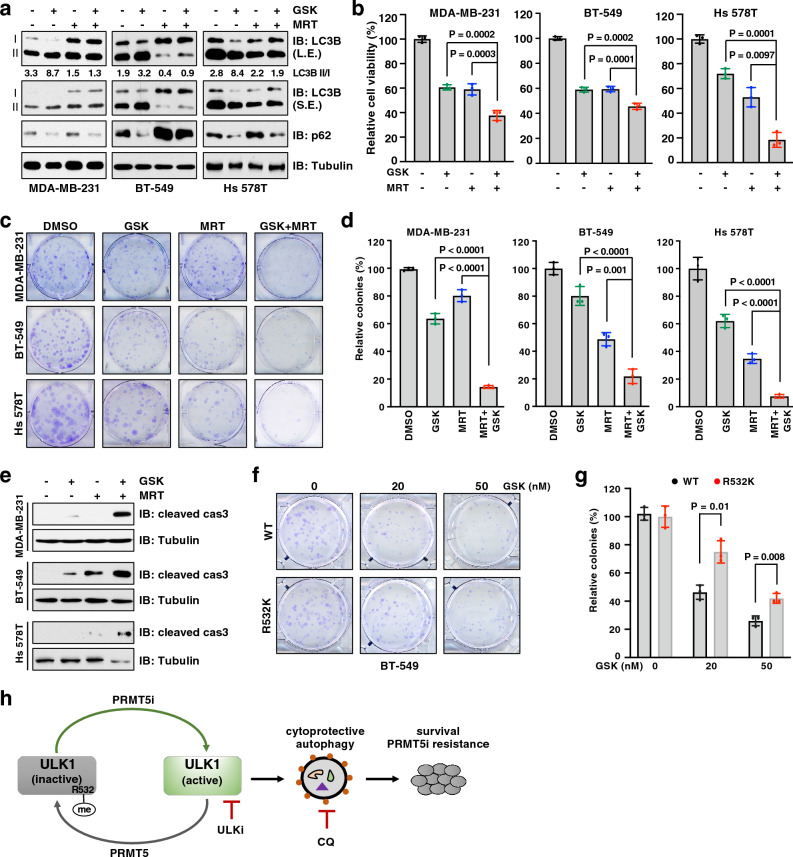
ULK1 inhibitor enhances cellular sensitivity to PRMT5 inhibitor. (**a**) IB analysis of WCL derived from indicated cells treated with 1 μM GSK3326595 (GSK) and 300 nM, 1.5 μM, and 0.5 μM MRT68921 (MRT, ULK inhibitor) for MDA-MB-231, BT-549, and Hs 578T respectively for 3 days before harvesting. (**b**) Cell viability of MDA-MB-231, BT-549, and Hs 578T cells after treatment with 1 μM GSK and 300 nM, 1.5 μM, and 0.5 μM MRT, respectively, for 4 days (MDA-MB-231 and BT-549) and 6 days (Hs 578T). (**c**,** d**) MDA-MB-231, BT-549 and Hs 578T cells were treated with DMSO,100 nM, 15 nM, and 50 nM GSK, respectively, and MRT concentration as described in (**b**). (**e**) IB analysis of WCL derived from indicated cells after treatment with 1 μM GSK and MRT as described in (**b**) for 3 days. (**f, g**) BT-549 cells were depleted of endogenous ULK1 and re-introduced inducible ULK1-WT or R532K. The resulting cells were treated with 0, 20, and 50 nM GSK and subjected to colony formation assays. (**h**) Graphical model to depict PRMT5 regulates autophagy by methylating ULK1 and targeting autophagy for overcoming resistance to PRMT5 inhibitor. PRMT5i, PRMT5 inhibitor; ULK1i, ULK1 inhibitor; CQ, chloroquine. In (**b**, **d**,** g**) data are shown as the mean ± SD of n = 3 independent experiments. *P* values were calculated by Student’s *t* test. Similar results were obtained in n = 3 independent experiments in (**a**, **e**).

Since ULK1 is a key druggable serine/threonine kinase for the induction of cytoprotective autophagy, targeting ULK1 therefore represents a promising therapeutic strategy for overcoming drug resistance ^[67]^. Having demonstrated that ULK1 plays a critical role in PRMT5-mediated autophagy regulation, we interrogated whether ULK1 inhibition would enhance sensitivity to PRMT5 inhibitor. Treatment with ULK1/2 inhibitor MRT68921^[68]^ largely suppressed GSK3326595-induced autophagy, as evidenced by a decrease of the LC3B II/I ratio and GFP-LC3B puncta (Fig. 6a and Supplementary Fig. 6a). As a result, combination of MRT68921 with GSK3326595 significantly decreased cell viability and colony formation in TNBC cells, compared to single agent (Fig. 6b–d). Moreover, apoptosis was strongly enhanced in cells treated with both GSK3326595 and MRT68921, compared to cells treated with single agent (Fig. 6e). Furthermore, cells expressing ULK-R532K displayed more colonies than cells expressing ULK-WT in the presence of GSK3326595 (Fig. 6f, g). These results suggest that ULK1 inhibition suppresses cytoprotective autophagy and consequently confers sensitivity to PRMT5 inhibitor in TNBC cells.

## Discussion

Over the past decade, extensive studies suggest that PRMT5 functions as an oncoprotein in various cancers through both epigenetic and non-epigenetic mechanisms^[11]^. Notably, PRMT5 is overexpressed in more than 50% of primary breast tumors and 70% of metastatic breast tumors, with strongest expression in TNBC^[15,29]^. These findings make PRMT5 as an attractive therapeutic target and pharmacological inhibition of PRMT5 represents a promising strategy for cancer therapy^[69]^. Our study demonstrates that PRMT5 inhibition evokes cytoprotective autophagy in part through promoting ULK1 activation, which sustains cell survival and confers resistance to PRMT5 inhibitors, and blockage of autophagy with ULK1 inhibitor or CQ remarkedly improve the efficacy of PRMT5 inhibitor in TNBC (Fig. 6h). Thus, our data establish a foundation for treatment of breast cancer with combinatorial inhibition of PRMT5 and autophagy.

PRMT5 is a versatile protein involved in many cellular processes^[70]^. Our finding revealed autophagy as another cellular process regulated by PRMT5. Although we showed that PRMT5 directly methylates ULK1 at R532 to suppress its kinase activity and basal autophagic function, we agree that ULK1-R532K mutant does not fully recapitulate the levels of autophagy induced by PRMT5 inhibition. It is possible that other mechanisms also contribute to PRMT5-mediated regulation of autophagy. For example, other ATG proteins and upstream autophagy modulators could be putative substrates of PRMT5. Indeed, PRMT5 have been documented to methylate and enhance AKT activation^[25]^, which negatively regulate autophagy by phosphorylating Beclin 1^[71]^. Moreover, PRMT5 is a crucial player in DNA damage response and DNA repair^[72]^, deficiency in which can induce autophagy^[73]^. These mechanisms may synergize with the defect in ULK1-R532 methylation to boost autophagy under condition of PRMT5 inhibition.

ULK1 functions as a conserved serine/threonine kinase in the autophagy pathway to sense upstream signals and initiate autophagy. During this process, PTMs, particularly phosphorylation, play a critical role in the dynamic regulation of the ULK1 activity^[74]^. Notably, by phosphorylating ULK1 at distinct residues of IDR, mTORC1 inhibits while AMPK activates autophagy in response to the changes of nutrition or energy in cells^[54,63]^. Our study demonstrates that PRMT5-mediated methylation of ULK1 at R532 reduces its kinase activity, adding another layer of ULK1 regulation regardless of the availability of nutrition. However, except for ubiquitination that has been shown to directly affect ULK1 stability^[75]^, the detailed mechanisms underlying how PTMs affects ULK1 activation have not yet been clearly established. Similarly, although it moderately affects ULK1 interaction with some of its substrates, ULK1-R532 methylation may also control ULK1 activity through other mechanisms. For example, ULK1-R532 methylation may cause its structurally conformational change or its interactions with other regulators, which warrants further studies.

While this manuscript was being prepared, a study reported that PRMT5/KDM5C-mediated dimethylation of ULK1 at R170 activates ULK1 to induce autophagy in LN229 glioblastoma (GBM) cells, Huh7 hepatocellular carcinoma (HCC) cells, and human oral keratinocytes (HOKs) in hypoxic environment, but not in normoxic condition^[76]^. However, it is unclear whether R170 is the sole site methylated by PRMT5 because they detected ULK1 arginine methylation only using the anti-ULK1-R170me2s antibody. Moreover, it is still needed to determine whether PRMT5 is involved regulation of autophagy under normoxic condition. By using the radioisotope-based in vitro arginine methylation assay, we demonstrated that R532 is the major methylation site by PRMT5. Our data also showed that PRMT5 depletion or PRMT5 inhibitor significantly induced autophagy in TNBC cells cultured in normal conditions. Therefore, PRMT5-mediated regulation of ULK1 activation and autophagy induction is likely dependent on environments and cell types.

## Methods

### Cell culture and reagents

All cells were obtained from American Type Culture Collection (ATCC). HEK293T, MDA-MB-231, MCF7, Hs 578T and their derived cell lines were maintained in Dulbecco’s modified Eagle’s medium (DMEM) (Genesee Scientific, 25–500). T-47D, MDA-MB-453, MDA-MB-468, BT-549, HCC70 and their derived cell lines were maintained in RPMI 1640 medium (Corning, 10-040-CV). 10%fetal bovine serum (FBS), 100 U/ml penicillin, and 100 μg/ml streptomycin were supplemented in the medium. GSK3326595 (HY-101563), MRT68921 dihydrochloride (HY-100006A), and Chloroquine (HY-17589A) purchased from MedChemExpress.

### Transfection, lentivirus production, and infection

For protein expression, transfection was performed using Lipofectamine 3000 (Thermo Fisher Scientific, L3000001) according to the manufacturer’s instructions. For lentivirus production, target constructs containing sgRNA or cDNA were co-transfected with packaging plasmids (pMD2G and pSPAX2) into HEK293T cells with Polyethylenimine (PEI, Polysciences, 23966-1). Twenty-four hours post transfection, fresh medium was replaced. Virus containing supernatants were harvested at 48 h post transfection and filtered with 0.45 μm PES filter. Targeted cells were infected with virus and selected with hygromycin (200 μg/ml), puromycin (1–2 μg/ml) or blasticidin (10 μg/ml) for 4 days to eliminate the non-infected cells.

### Plasmids

Flag-PRMT5, Flag-MEP50 were generated by cloning the corresponding cDNA into the pRK5-Flag vector while HA-PRMT5 and HA-ULK1 cDNA were cloned into the pRK5-HA vector. Myc-PRMT5, Myc-ULK1, Myc-Beclin 1, and Myc-Ambra1 were generated by cloning the corresponding cDNA into the pRK5-Myc vector. GST-Beclin 1 (1–86 aa) and GST-ULK1 (1–649 aa) were generated by inserting the cDNA into pGEX-6P-1 bacteria expression vector. Myc-ATG13 (#31965), Flag-FIP200 (#24300), GFP-LC3-RFP (#84573) were purchased from Addgene. Lentiviral HA-ULK1 and HA-PRMT5 were generated by cloning the corresponding cDNA into pLenti-HA-hygro vector or pLJM1-HA-puro vector. PRMT5-E444Q, ULK1-R532K and various ULK1 mutants were generated using the QuikChange XL site-directed mutagenesis kit. Various single guide RNAs (sgRNA) were designed at https://www.synthego.com and were cloned into lentiCRISPR v2 vector (Addgene, #52961). Sequence of sgRNAs is listed Supplementary Table 1.

### Antibodies

All primary antibodies were diluted with 5% non-fat milk in TBST buffer for Western blot. Anti-ULK1 (8045), anti-Myc (2278), anti-cleaved Caspase 3 (9661), anti-AMPKα (5831), anti-Raptor (2280), anti-pS757-ULK1 (14202), anti-pS15-Beclin 1 (84966), anti-LC3B (3868), anti-ATG13 (13468), anti-PRMT1 (2449), anti-PRMT5 (79998), anti-S6K1 (9202), anti-HA (3724), and anti-pT389-S6K (9234) were purchased from Cell Signaling Technology. Anti-Tubulin (66240-1-lg) and anti-Beclin 1 (11306-1-AP) were purchased from Proteintech. Rabbit anti-FLAG (F7425), mouse anti-FLAG (F3165), peroxidase-conjugated anti-mouse secondary antibody (A4416), and anti-rabbit secondary antibody (A4914) were purchased from Sigma. Monoclonal anti-HA (901503) was purchased from BioLegend. Anti-PRMT7 (A12159) and anti-p62 (A11483) were purchased from ABclonal. Anti-pS318-ATG13 (600-401-C49) was purchased from ROCKLAND. Anti-MMA was a gift from Dr. Mark Bedford at MD Anderson Cancer Center.

### Immunoblot (IB) and immunoprecipitation (IP) analyses

Cells were rinsed with ice-cold phosphate-buffered saline (PBS) and lysed in EBC buffer (50 mM Tris–HCl pH 7.5, 120 mM NaCl and 0.5% NP-40) or Triton buffer (40 mM HEPES pH 7.4, 150 mM NaCl, 2.5 mM MgCl_2_, 1 mM EDTA and 1% Triton X-100) supplemented with protease inhibitor (Thermo Fisher, A32953) and phosphatase inhibitors (phosphatase inhibitor cocktail Set I and II, Calbiochem). The cell lysates were centrifuged at 13,200 r.p.m. at 4 °C for 10 min. The protein concentration of lysates was determined using Nanodrop by Bio-Rad protein assay reagent. Equal amounts of whole cell lysates were resolved by SDS-PAGE and immunoblotted with indicated antibodies. For IP, 2000–5000 μg lysates were incubated with agarose conjugated antibodies for 3–5 h at 4 °C. Immunoprecipitants were washed three times with NETN buffer (20 mM Tris–HCl, pH 8.0, 150 mM NaCl, 1 mM EDTA and 0.5% NP-40) or Triton buffer before being resolved by SDS-PAGE. Anti-HA agarose beads (A2095) and anti-FLAG agarose beads (A2220) were purchased from Sigma-Aldrich. Anti-Myc agarose beads (658502) were purchased from BioLegend. Some blots were cut prior to hybridization with primary antibodies, but one full-length original, unprocessed blot for each antibody was provided in the Supplementary Materials.

### Purification of GST-tagged protein from *E. coli*

Recombinant GST-ULK1 and GST-Beclin 1 truncated proteins were purified from the BL21(DE3) Escherichia coli transformed with corresponding constructs. Single colony was grown in 7 mL Luria–Bertani (LB) medium overnight at 37 °C. The culture was then inoculated into 400 mL LB medium until an optical density of 0.5–0.6. The protein expression was induced by 0.1 mM IPTG (isopropyl-β-D-thiogalactoside) at 25 °C for 16 h. The bacteria cells were collected and re-suspended in GST buffer [25 mM Tris–HCl pH 8.0, 5 mM dithiothreitol (DTT), 150 mM NaCl] and sonicated. After centrifugation, the supernatant was incubated with glutathione sepharose beads for 3 h at 4 °C. The protein-bound glutathione beads were washed three times with GST buffer and recombinant GST proteins were eluted with elution buffer (10 mM L-Glutathione, 50 mM Tris–HCl pH 8.0).

### In vitro methylation assays

3 μg recombinant GST-ULK1 truncated proteins were incubated with HA-PRMT5/MEP50 in the methylation buffer (50 mM Tris–HCl pH 8.5, 20 mM KCl, 10 mM MgCl_2_, 1 mM β-mercaptoethanol, 100 mM sucrose) with 1 μL of adenosyl-L-methionine, S-[methyl-3H] (1 mCi/ml stock solution, Perkin Elmer) at 30 °C for 1 h. The reactions were stopped by 3 × SDS loading buffer. The samples were resolved by SDS-PAGE and transferred to PVDF membrane, which was then sprayed with EN3HANCE (Perkin Elmer) and exposed to X-ray film.

### In vitro kinase assays

3 μg of bacterially purified GST-Beclin 1 recombinant proteins were incubated with HA-ULK1 purified from HEK293T cells in the kinase reaction buffer (25 mM HEPES pH 7.4, 50 mM NaCl, 5 mM MgCl_2_, 0.1 mM DTT, 0.5 mg/ml BSA) for 30 min at 30 °C. The reaction was stopped by adding 2 × SDS loading buffer. Samples were incubated at 100 °C for 5 min and resolved by SDS-PAGE. Phosphorylation of GST-Beclin 1 was detected by anti-pS15-Becllin 1 antibody.

### Immunofluorescence staining

Cells grown on glass coverslips were fixed with 4% paraformaldehyde for 15 min at room temperature, washed three times with PBS, and then permeabilized with 0.05% Triton X-100 for 10 min at room temperature. Following three washes with PBS, cells were stained with DAPI, washed four times with PBS and mounted using vibrance antifade mounting medium (Vector Laboratories, H-1700). Images were taken by Leica SP8 Confocal microscope and puncta were counted manually.

### Mass spectrometric analysis of ULK1-R532 methylation

HEK293T cells were transfected with HA-ULK1. Forty-eight hours post transfection, the cells were lysed in Triton buffer, followed by immunoprecipitation. The immunoprecipitates were resolved by SDS-PAGE and visualized using GelCode blue staining reagent (Thermo Scientific, 24590). The protein band containing HA-ULK1 was excised and digested with trypsin. Peptides were analyzed on an EASY nLC 1200 in-line with the Orbitrap Fusion Lumos Tribrid mass spectrometer (ThermoScientific). Peptides were pressure loaded at 800 bar and separated on a C18 reversed phase column (Acclaim PepMap RSLC, 75 μm × 50 cm (C18, 2 μm, 100 Å)) (Thermo Fisher) using a gradient of 2–35% B in 180 min (Solvent A: 0.1% FA; Solvent B: 80% ACN/0.1% FA) at a flow rate of 300 nL/min at 45 °C. Mass spectra were acquired in datadependent mode with a high resolution (60,000) Fourier Transform mass spectrometry (FTMS) survey scan followed by MS/MS of the most intense precursors with a cycle time of 3 s. The automatic gain control target value was 4.0e5 for the survey MS1 scan. Precursors were isolated with a 1.6 *m/z* window with a maximum injection time of 50 ms. Tandem mass spectra were acquired using higher-energy collisional dissociation (HCD) and electron transfer dissociation (ETD) for each peptide precursor in an alternating fashion. The HCD collision energy was 35% and ETD was performed using the calibrated charge dependent ETD parameters. The fragment ions were detected in the Orbitrap at 15,000 resolution. Spectra were searched against a custom database containing human ULK1 and a database of common contaminants using MaxQuant and Proteome Discoverer. The false discovery rate, determined using a reversed database strategy, was set at 1% at the peptide and modification site levels. Fully tryptic peptides with a minimum of seven residues were required including cleavage between lysine and proline. Two missed cleavages were permitted. Sites of modification were manually verified.

### Cell viability assays

Cells were seeded in 96-well plate at 500–1000 cells per well for 24 h and then treated with indicated doses of inhibitors for 4 days. Cell viability was determined using the Cell Titer-Glo cell viability assay kit according to the manufacturer’s instructions (Promega, G7570).

### Clonogenic survival assays

Cells were seeded in 6-well plates at 300–500 cells per well for 24 h and then treated with indicated inhibitors for 8–10 days until visible colonies formation. Fresh medium with inhibitors was replaced every 3 days. Colonies were fixed with 10% ethanol and 10% acetic acid for 30 min and then stained with 0.4% crystal violent in 20% ethanol for 30 min, followed by wash with dH2O and manually counted.

### Statistical analysis

As indicated in the figure legends, all quantitative data are presented as the mean ± SD of three biologically independent experiments or samples. Statistical analyses were performed using GraphPad Prism 9 and Excel. Statistical significance was determined by two-tailed Student’s *t* test or two-way ANOVA. *P* value < 0.05 was considered significant.

## Supplementary Information

Supplementary Information.

## Data Availability

All data generated or analyzed during this study are included in this published article and its supplementary information files.
