# Supplementary material for: Autophagy dictates sensitivity to PRMT5 inhibitor in breast cancer
Source: Sci Rep. 2023 Jul 3;13:10752. doi: 10.1038/s41598-023-37706-9 (PMC10318021; doi:10.1038/s41598-023-37706-9)

Images of the original blots for Fig. 2

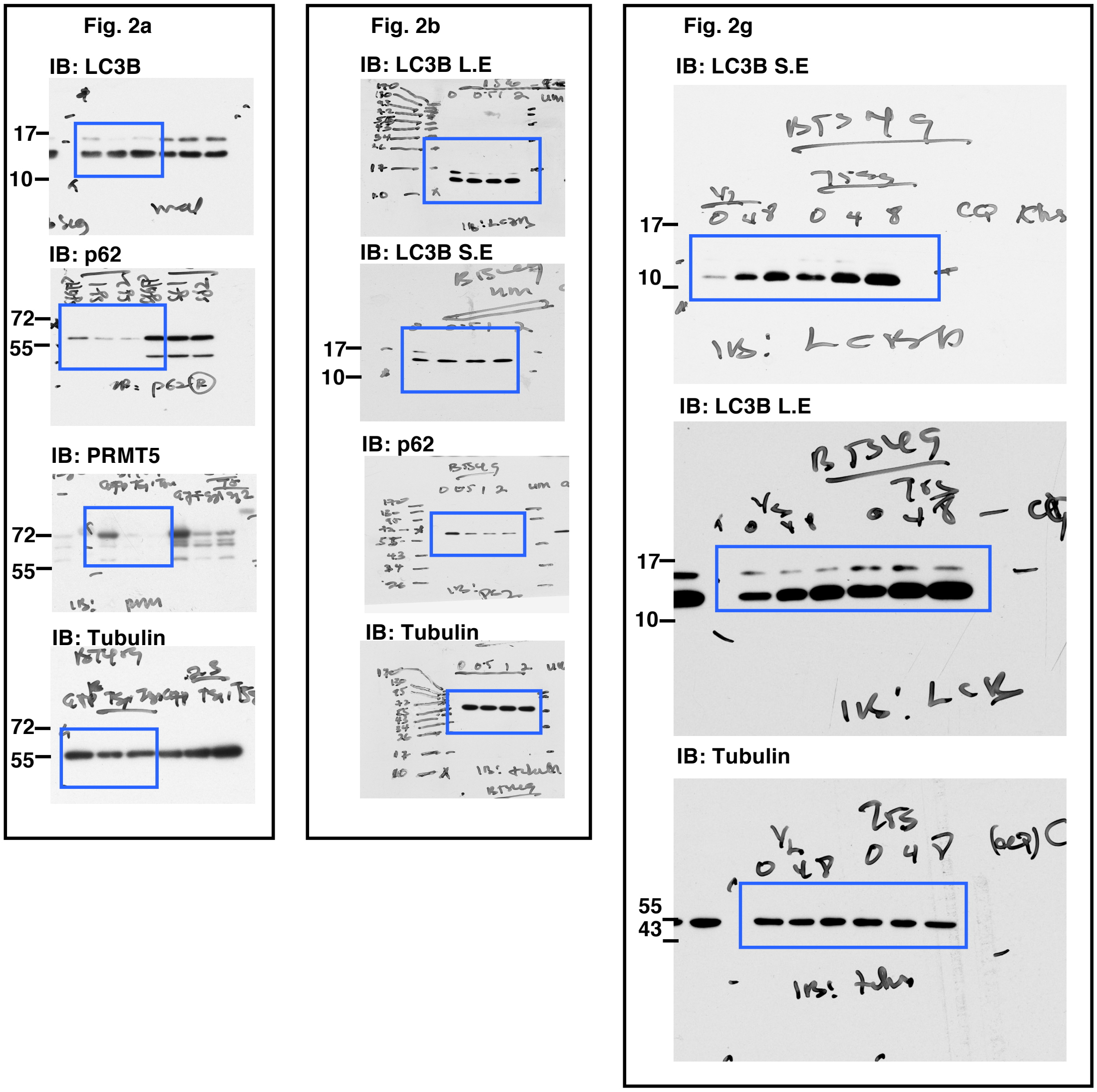

Images of the original blots for Fig. 3

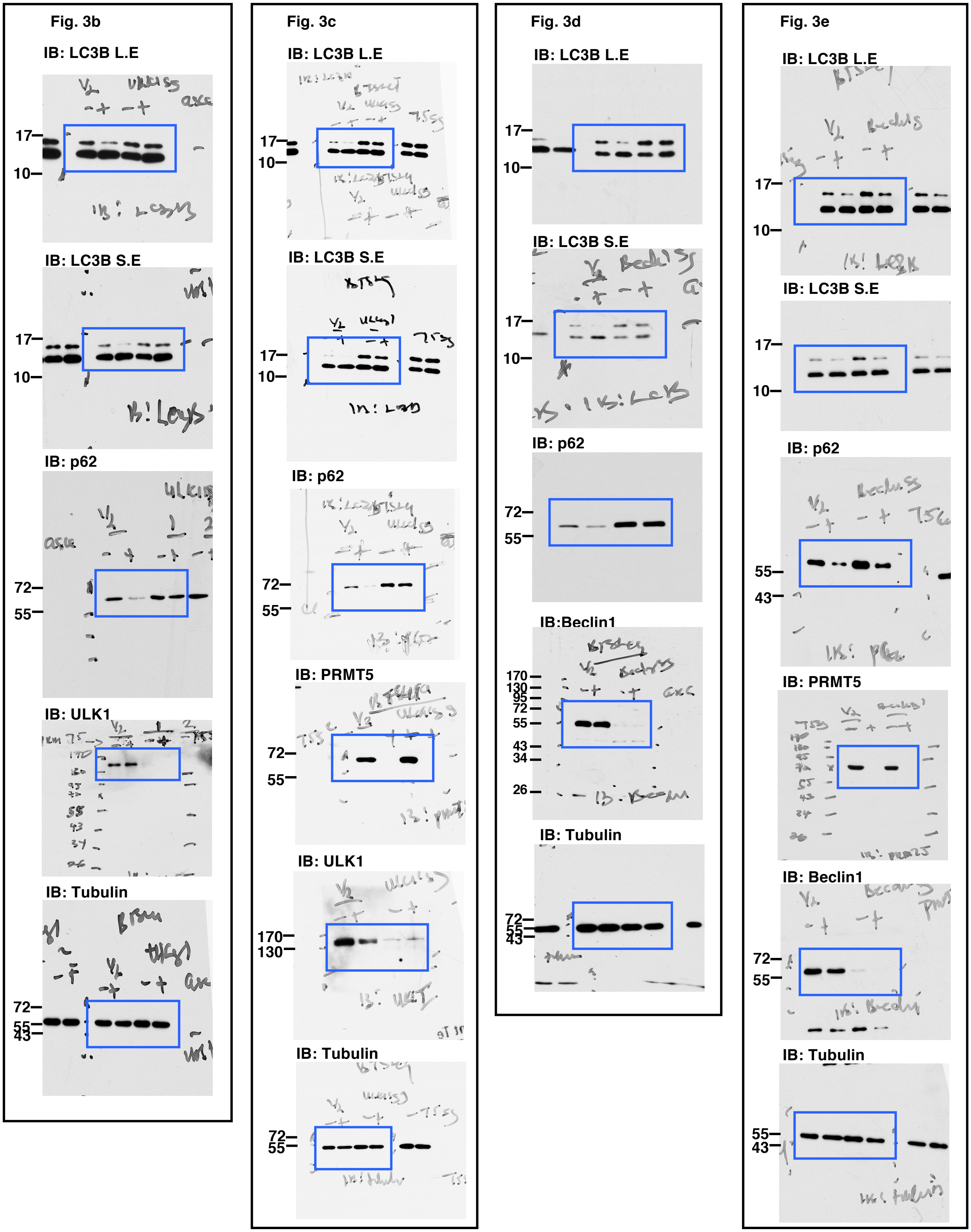

Images of the original blots for Fig. 4

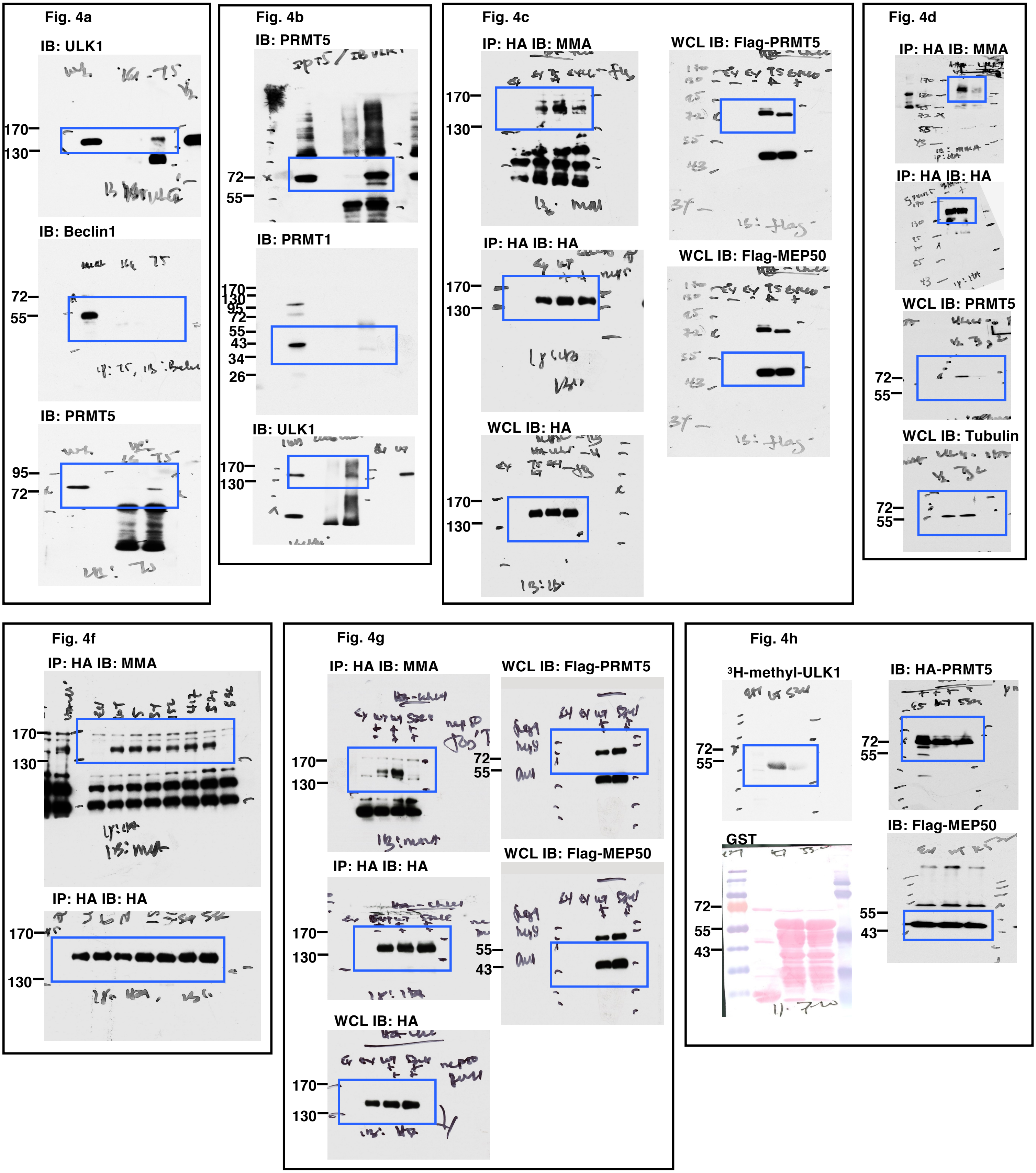

Images of the original blots for Fig. 5

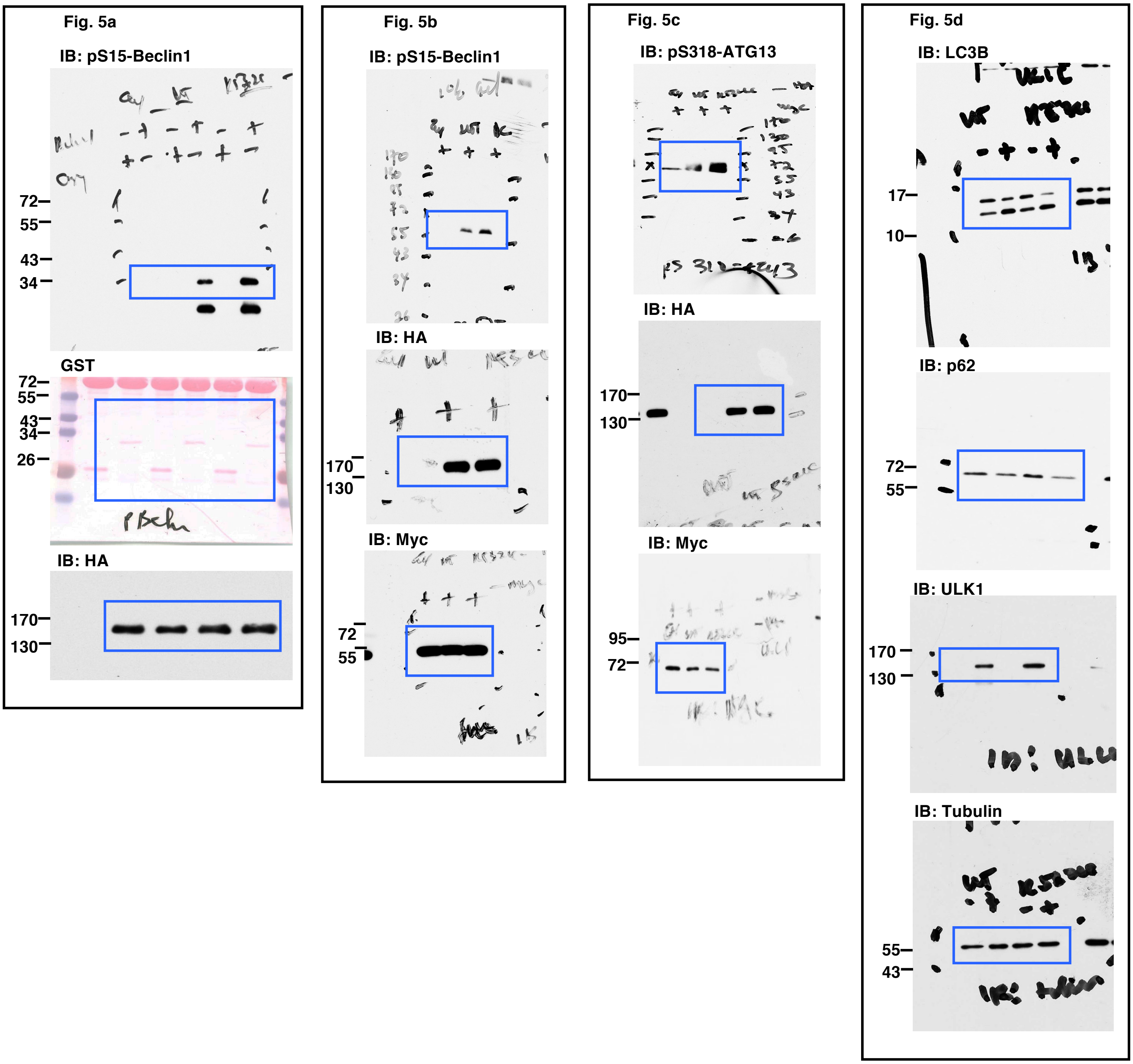

Images of the original blots for Fig. 6

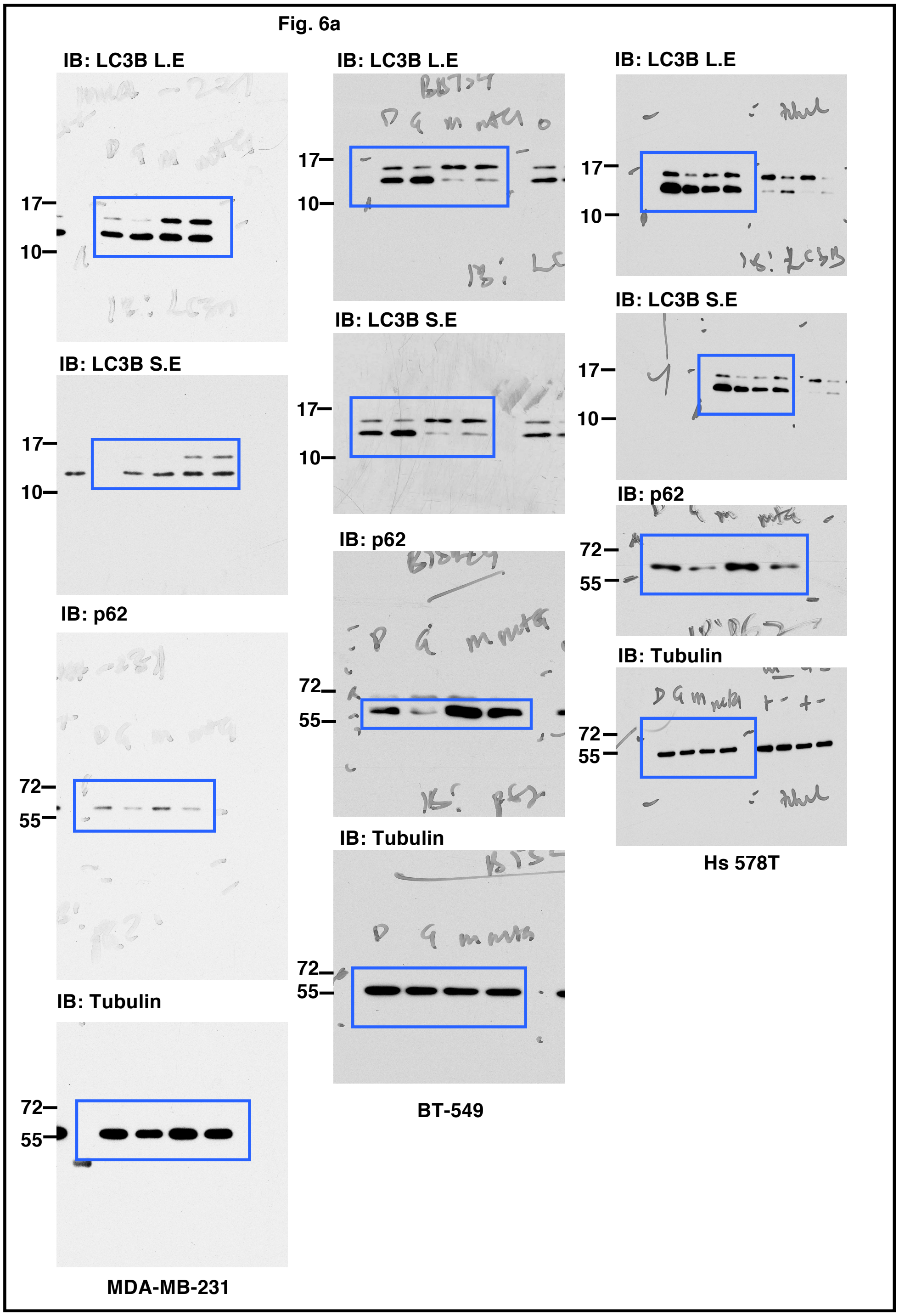

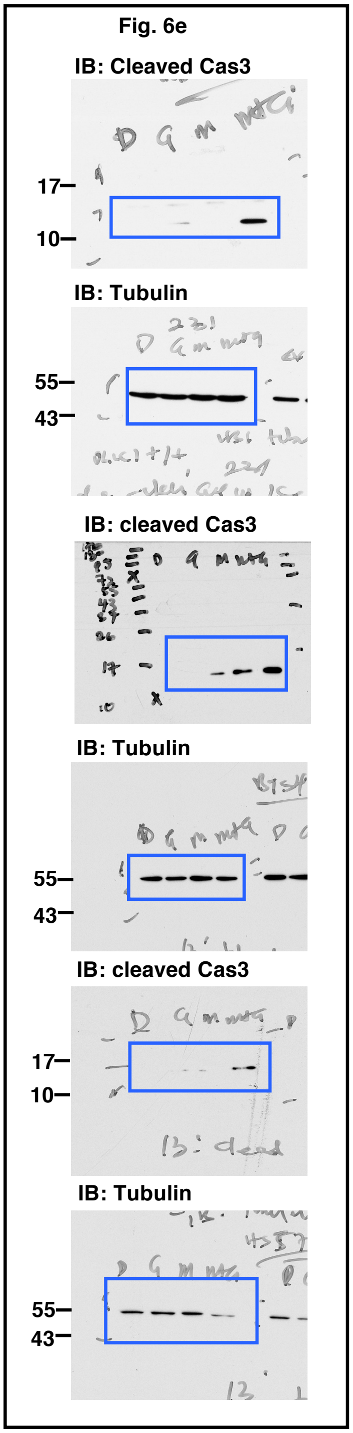

Images of the original blots for Suppl Fig. 1

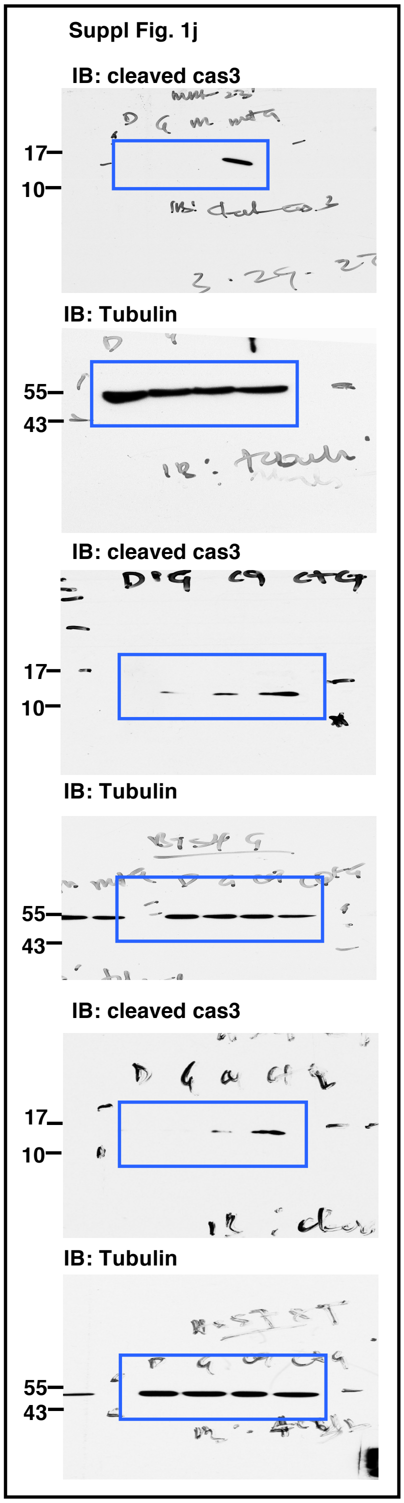

Images of the original blots for Suppl Fig. 2

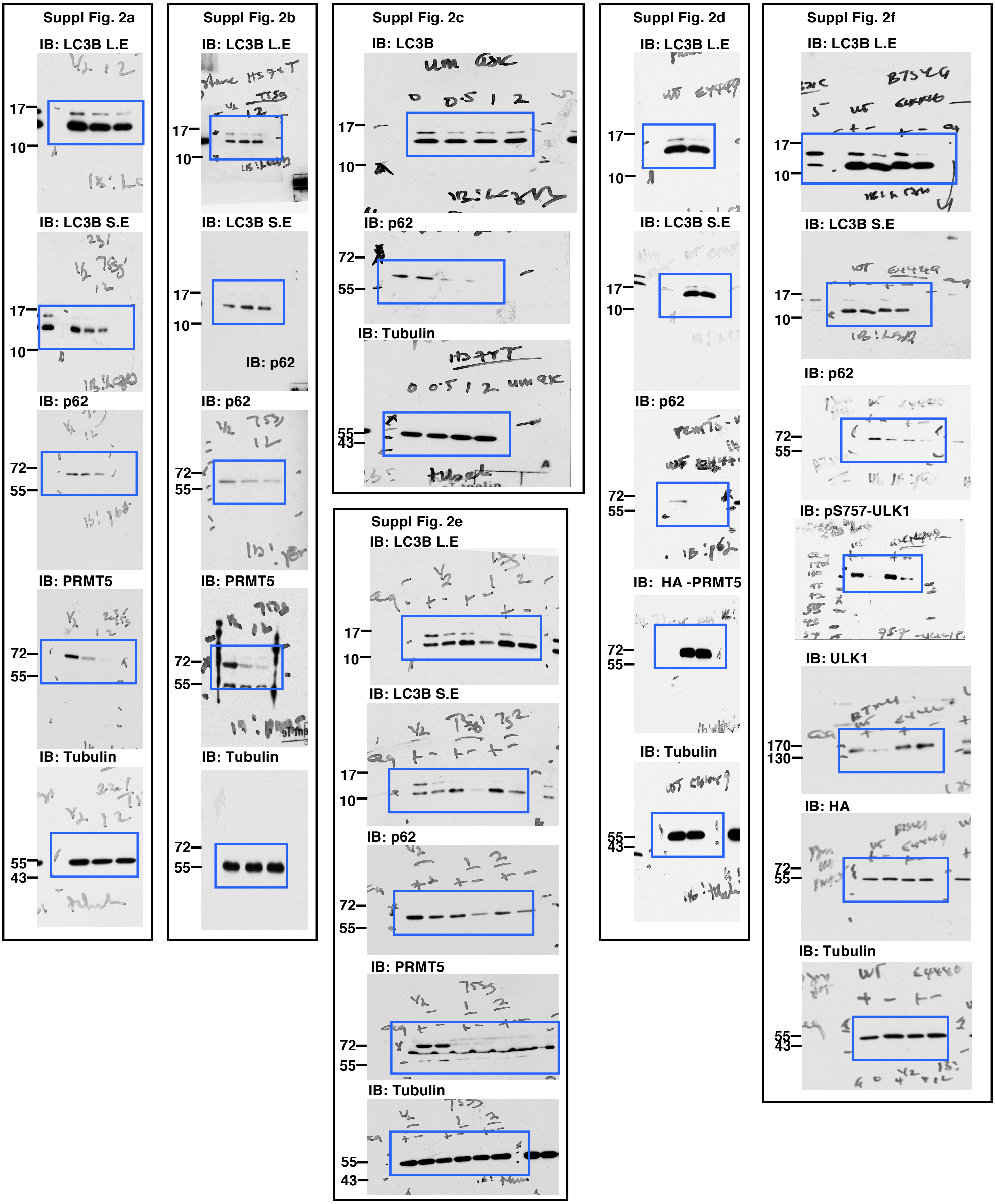

Images of the original blots for Suppl Fig. 3

Suppl Fig. 3

IB: LC3B

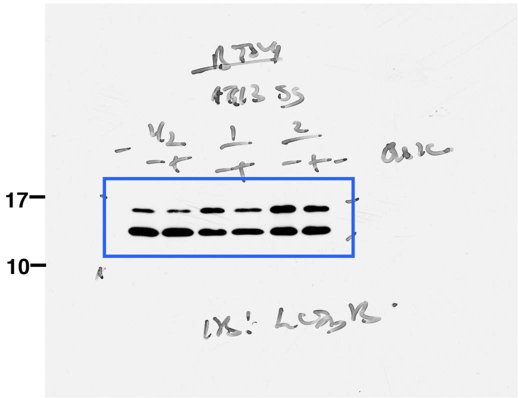

IB:p62

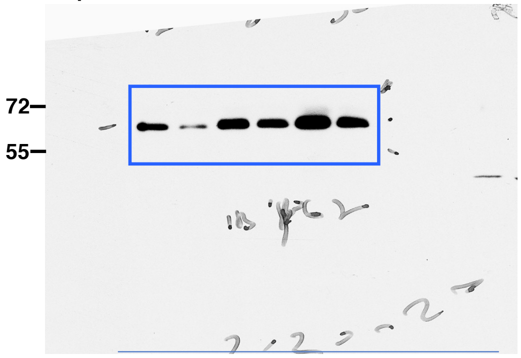

IB: ATG13

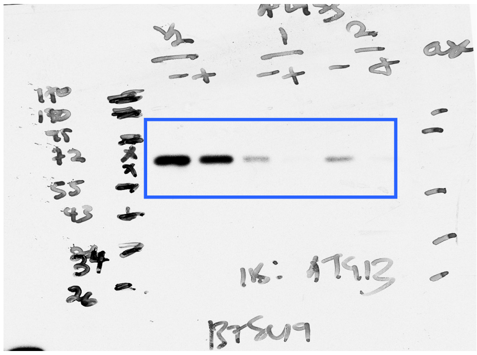

IB: Tubulin

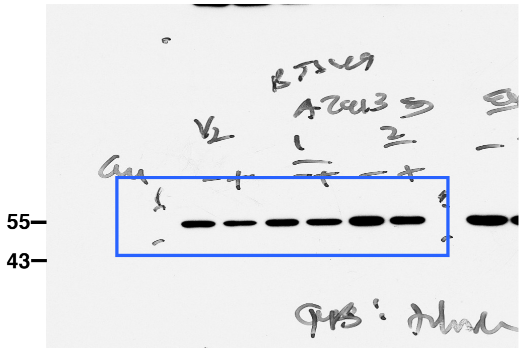

Images of the original blots for Suppl Fig. 4

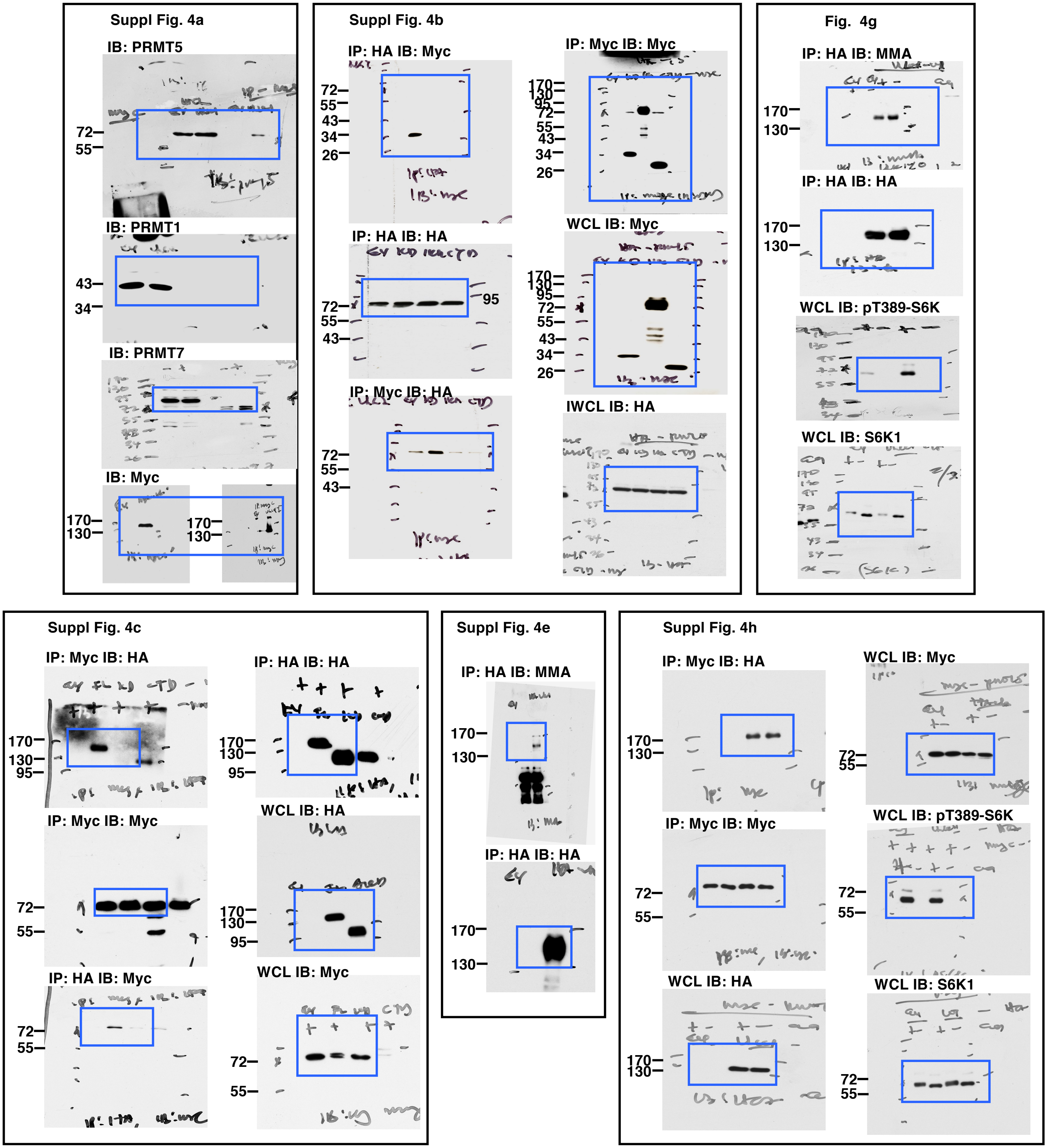

Images of the original blots for Suppl Fig. 5

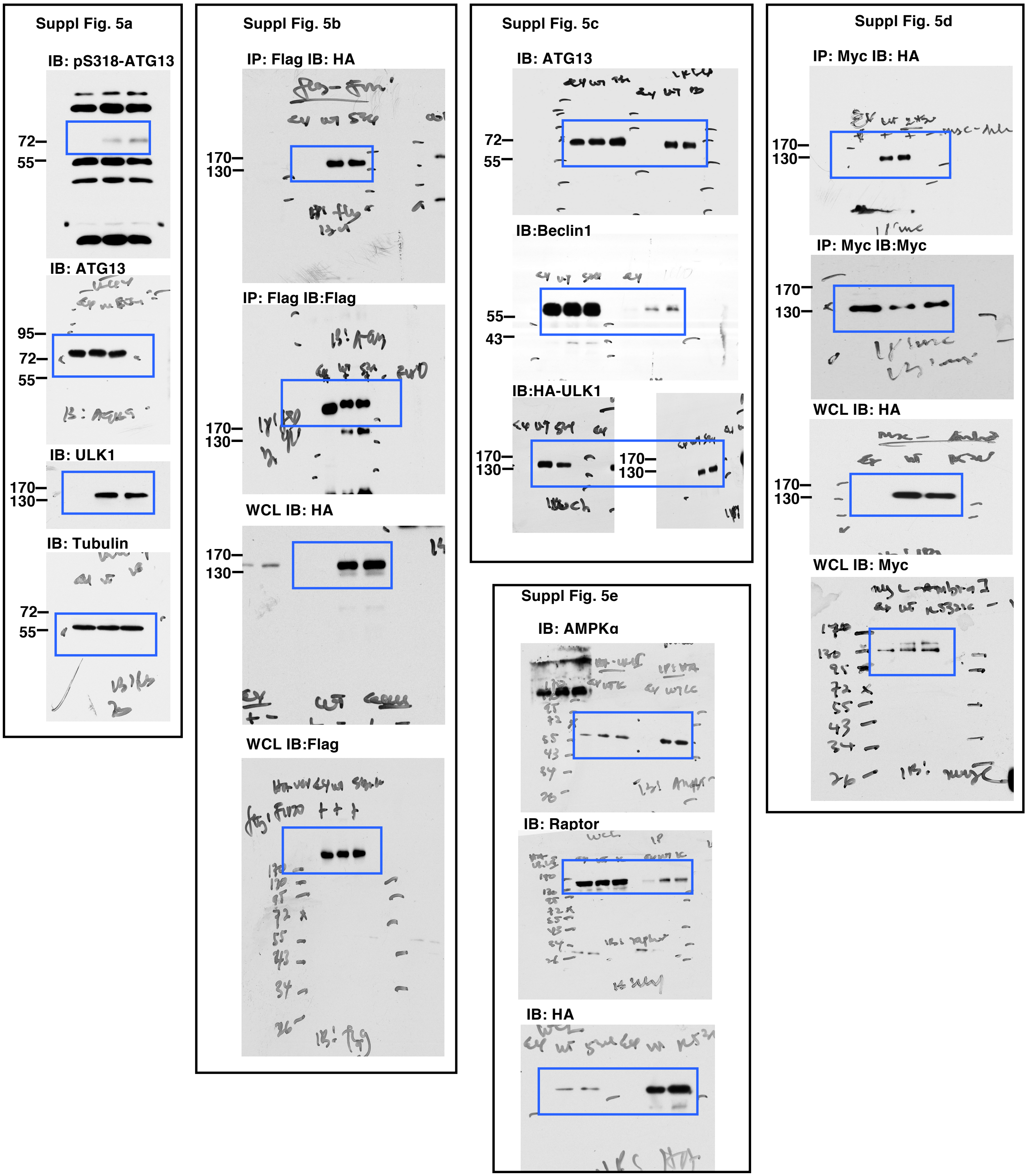

Supplement: Supplementary file 1 — Supplementary Information. [file 41598_2023_37706_MOESM1_ESM.pdf]
